# Adaptation of yeast *Saccharomyces cerevisiae* to grape-skin environment

**DOI:** 10.1038/s41598-023-35734-z

**Published:** 2023-06-20

**Authors:** Daisuke Watanabe, Wataru Hashimoto

**Affiliations:** 1 Laboratory of Basic and Applied Molecular Biotechnology, Division of Food Science and Biotechnology, Graduate School of Agriculture, Kyoto University, Uji, Kyoto Japan; 2 Laboratory of Applied Stress Microbiology, Division of Biological Science, Graduate School of Science and Technology, Nara Institute of Science and Technology, Ikoma, Nara, Japan

**Keywords:** Microbiology, Microbial communities, Fungi

## Abstract

*Saccharomyces cerevisiae*, an essential player in alcoholic fermentation during winemaking, is rarely found in intact grapes. Although grape-skin environment is unsuitable for *S. cerevisiae*’s stable residence, Saccharomycetaceae-family fermentative yeasts can increase population on grape berries after colonization during raisin production. Here, we addressed adaptation of *S. cerevisiae* to grape-skin ecosystem. The yeast-like fungus *Aureobasidium pullulans*, a major grape-skin resident, exhibited broad spectrum assimilation of plant-derived carbon sources, including ω-hydroxy fatty acid, arising from degradation of plant cuticles. In fact, *A. pullulans* encoded and secreted possible cutinase-like esterase for cuticle degradation. When intact grape berries were used as a sole carbon source, such grape-skin associated fungi increased the accessibility to fermentable sugars by degrading and assimilating the plant cell wall and cuticle compounds. Their ability seems also helpful for *S. cerevisiae* to obtain energy through alcoholic fermentation. Thus, degradation and utilization of grape-skin materials by resident microbiota may account for their residence on grape-skin and *S. cerevisiae*’s possible commensal behaviors. Conclusively, this study focused on the symbiosis between grape-skin microbiota and *S. cerevisiae* from the perspective of winemaking origin. Such plant–microbe symbiotic interaction may be a prerequisite for triggering spontaneous food fermentation.

## Introduction

The origin of wine yeasts, taxonomically categorized into *Saccharomyces cerevisiae* or its closely related species, has been an issue of great controversy. Historically, wine yeasts were first found on grape surfaces^1^, whereas *Saccharomyces* species are absent or rare in fresh grape berries^2–4^. Goddard and Greig propose a neutral nomad model for *S. cerevisiae*, since there is very little direct evidence for its adaptation to fruit or any other specific niche^5^. Studies based on high-throughput sequencing technologies have revealed the grape microbiota including bacteria and fungi^6–9^. Regardless of geographical and cultivar features, sound grapes share a core fungal microbiota consisting of the yeast-like ascomycetous fungi *Aureobasidium pullulans*, basidiomycetous yeasts, and other ascomycetous non-*Saccharomyces* yeasts^6–9^, suggesting a microbial strategy to selectively adapt to grape and vineyard environments. *S. cerevisiae*, even if detected, constitutes only a very minor part of the microbial community. Assuming that *Saccharomyces* yeast species are transferred from soil or vectored by insects or migratory birds^10,11^, the first colonist wine yeasts must endure and adapt in vineyard environments, nutritionally undesirable for survival and proliferation. Possible benefits to *S. cerevisiae* through interactions with the core grape microbiota are of our special interest.

*S. cerevisiae* wine yeast cells convert fermentable sugars of grapes (e.g., glucose, fructose) into energy via alcoholic fermentation. In contrast, fungal resident microbiota in intact grapes are predominantly irrelevant to winemaking due to their lack of alcoholic fermentation ability^3,4,6,8^. The key grape-skin components required for colonization and adaptation of the grape microbiota remain elusive. If grape-inhabiting microorganisms mediate wine yeast’s adaptation, they might somehow promote alcoholic fermentation for *S. cerevisiae* cells to obtain energy. Nevertheless, previous reports on coculture of *A. pullulans* or basidiomycetous yeast with *S. cerevisiae*^12–14^ have no mention of positive effects on ethanol production from grape juice during winemaking.

The grape-skin, the natural habitat for non-*Saccharomyces* oligotrophic microorganisms, covers and protects the pulp for nutrient storage^15,16^. The grape-skin occupies approximately 10% of the dry weight of grape berries and acts as a barrier against dehydration, physical damage, and microbial penetration. The plant primary cell wall components, cellulose, hemicellulose, and pectin, contribute to the structural integrity. The grape-skin is particularly abundant in pectinic acid, the methyl-esterified form of polygalacturonic acid^17,18^. As previously reported, many grape-inhabiting microorganisms secrete cellulase, pectinase, and relevant degrading enzymes to use the cell wall decomposition products as nutrients^19–22^. The microbial targeting ability to degrade and assimilate the plant cell wall compounds is likely a prerequisite for colonization and adaptation to vineyard environments. Wine yeasts of the *Saccharomyces* genus may need the aid of grape-skin residents to survive on the surface of grapes due to the absence of degrading or metabolizing enzymes for plant cell wall and decomposition products^23,24^.

The outermost layer of grape-skin is the cuticle, mainly composed of the lipid polyester cutin (i.e., ester-linked ω-hydroxy C_16_ and C_18_ fatty acids) and the chemically highly resistant biopolymer cutan^25,26^. On all aerial surfaces of land plants, thick and hydrophobic cuticle layers prevent desiccation, UV damage, and pathogen infection. At the frontline, the plant cuticle also serves multifunctional roles in triggering immunity during plant-pathogen interactions^27^. Thus, cuticle-degrading virulent pathogens can cause severe damage to terrestrial plants. The cutin-hydrolyzing enzyme cutinase has been extensively studied in typical plant pathogens of *Fusarium* species and in a limited number of molds, yeasts, and bacteria^28–30^. However, the degradation and assimilation of the plant cuticle compounds by non-pathogenic, grape-skin microorganisms are yet to be described.

This study proposed a novel tripartite relationship between grape berries, grape-skin microbiota, and *S. cerevisiae* from the viewpoint of alcoholic fermentation. Similar plant-microbial interactions may typically occur in traditional production processes of fermented foods and beverages, where humans have optimized the growth of characteristic microbial communities over thousands of years^31^. Thus, this study provides an important clue to understand the dynamics and mechanism of plant-microbial ecosystems by analyzing the origin of wine fermentation as an experimentally tractable model.

## Results

### Interaction between grape-skin microbiota and *S. cerevisiae* on raisins

**Figure 1:**
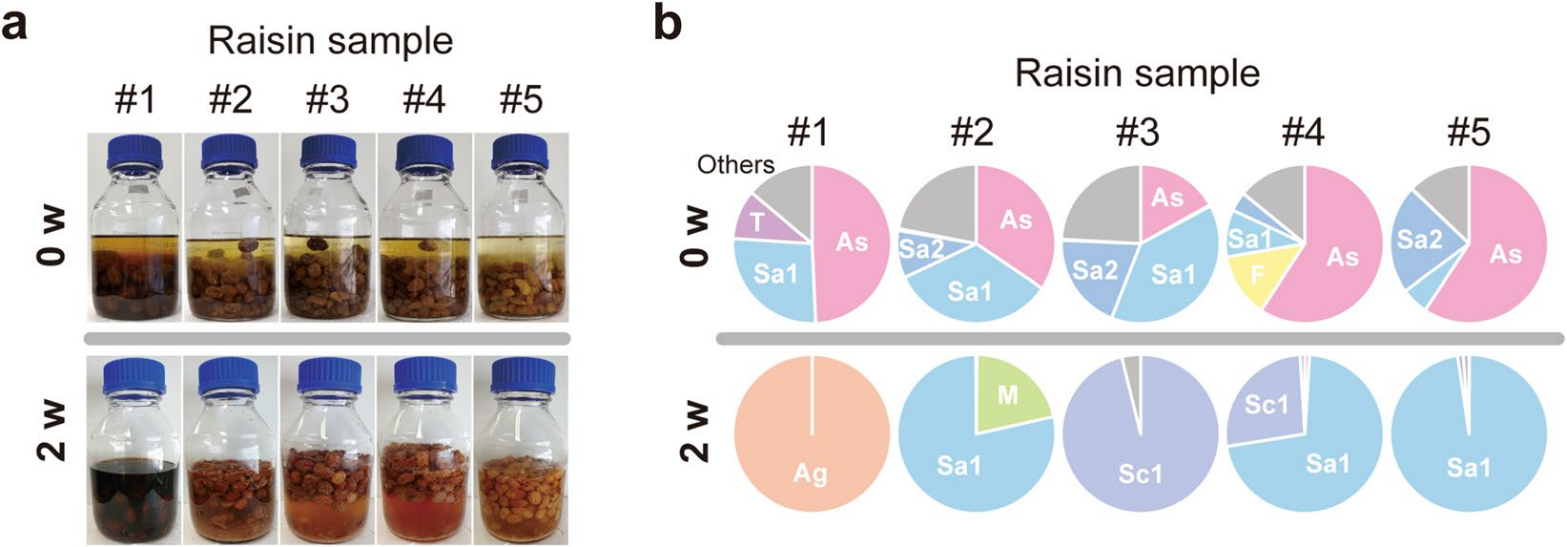
Fungal microbiota in raisins and raisin yeast. (**a**) Raisin-yeast making using five different industrial raisin samples. Raisins were mixed with water and incubated until fermentation takes place. (**b**) ITS-based profiling of fungal microbiota composition at the family level on the surface of raisins (0 w) and in raisin yeast (2 w). Ag, Agaricomycetes (class; family unidentified); As, Aspergillaceae; F, Filobasidiaceae; Sa1, Saccharomycetaceae; Sa2, Saccharomycodaceae; Sc1, Schizosaccharomycetaceae; T, Trichosporonaceae.

To investigate *S. cerevisiae*’s behavior on grape-skin environments, we focused on raisin microbiota. Although *S. cerevisiae* is rarely found from sound grape berries^2–4^, raisins are traditionally and commonly used as a source of dough-fermenting *S. cerevisiae* in homemade bread making^32^. Commercially available raisins from different manufacturers were covered with water and statically incubated (Fig. 1a). In all experiments but sample #1, raisins floated, the liquid turned cloudy, and numerous small bubbles were observed within a few days. This is what is called “raisin water” or “raisin yeast”. As shown in Fig. 1b, fungal internal transcribed spacer (ITS) amplicon sequence analysis indicated that common predominant fungal families in all raisin samples were (i) Aspergillaceae, composed of *Aspergillus* mold species, (ii) Saccharomycetaceae, including *S. cerevisiae* and its relative species (e.g. *Zygosaccharomyces rouxii*, *Lachancea dasiensis*), and (iii) Saccharomycodaceae, mainly *Hanseniaspora uvarum*, which is one of the most abundant yeast species at the beginning of natural fermentations. After two-week incubation, raisin yeast from samples #2–#5 was exclusively occupied by Saccharomycetaceae and Schizosaccharomycetaceae family fermentative yeast species. Notably, initial yeast species on raisins do not always propagate in raisin yeast, suggesting complicated fungal interaction and succession. Although sample #1 contained plenty of *S. cerevisiae*, raisin yeast making failed. Thus, the raisin production process is the primary step for *S. cerevisiae* and other fermentative yeasts to trigger colonization into grape environments.

**Figure 2:**
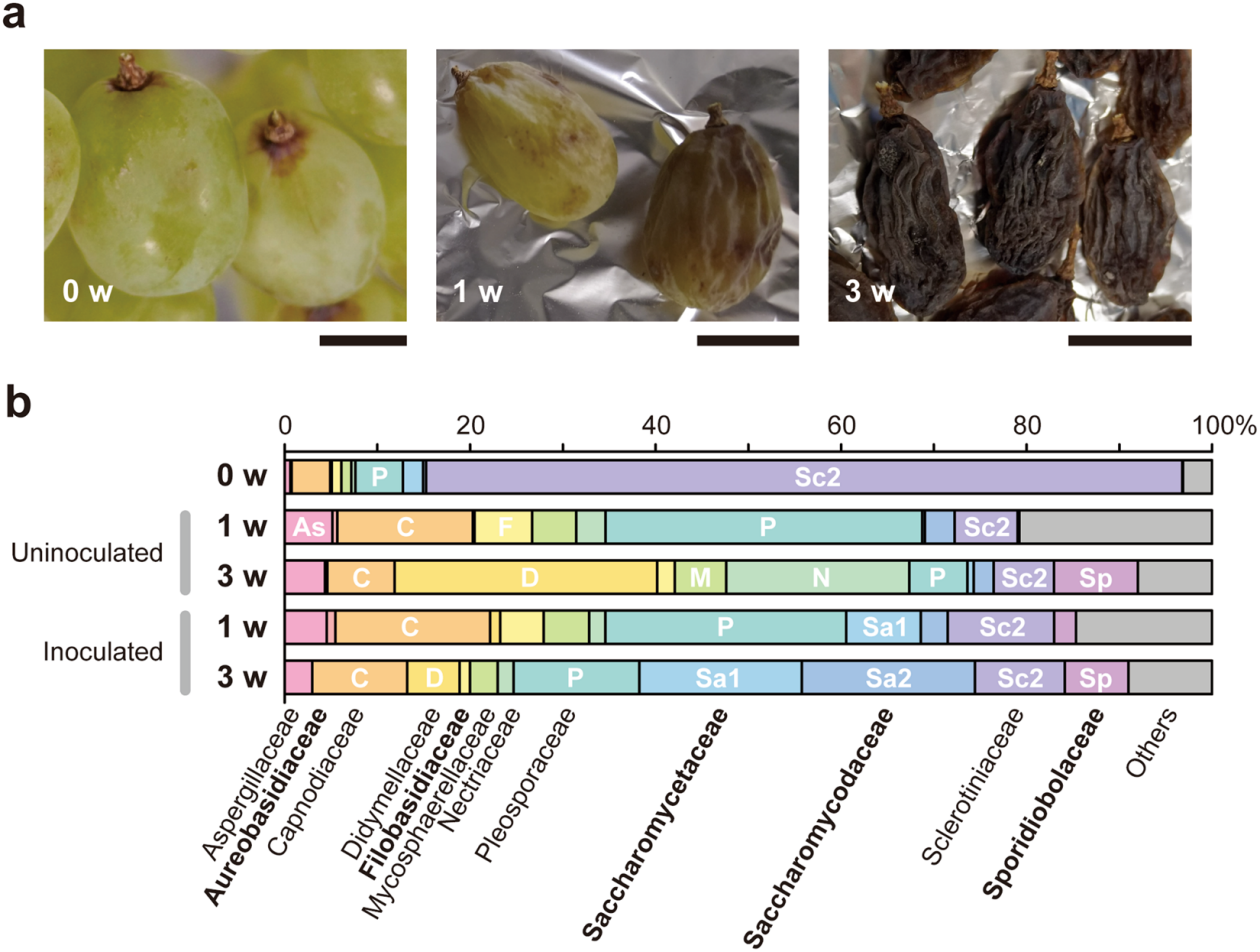
Fungal microbiota during raisin production. **(a**) Drying grape berries. Bars, 1 cm. (**b**) ITS-based profiling of fungal microbiota composition at the family level on the surface of drying grape berries. Families with a relative frequency exceeding 5% are labelled as white: As, Aspergillaceae; C, Capnodiaceae; D, Didymellaceae; F, Filobasidiaceae; M, Mycosphaerellaceae; N, Nectriaceae; P, Pleosporaceae; Sa1, Saccharomycetaceae; Sa2, Saccharomycodaceae; Sc2, Sclerotiniaceae; Sp, Sporidiobolaceae. Yeast families are indicated in bold.

We aseptically dried sound grape berries in an experimental oven for metagenomic analysis during the raisin production process (Fig. 2a). The berries discolored, shriveled, and eventually developed raisin-like wrinkles within 3-week fan drying process. In spite of repeated trials, we failed to detect *S. cerevisiae* from resulted raisins (Fig. 2b). In most cases, Sclerotiniaceae family molds (e.g., *Botrytis* a.k.a. “noble rot”) dominated fresh berries and drastically decreased during the drying process. In the representative experiment shown in Fig. 2b, after fungal families such as Aspergillaceae, Capnodiaceae, and Pleosporaceae transiently increased the frequency, Didymellaceae, Nectriaceae, and Sporidiobolaceae dominated. Rarely detected yeast species from Saccharomycetaceae family were almost non-*Saccharomyces* (e.g., *Candida*, *Pichia*) and did not increase the population. In contrast, inoculation of *S. cerevisiae* X2180 cells (approximately 10 cells/berry) on the surface of grape-skin before drying resulted in the increased abundance of Saccharomycetaceae and Saccharomycodaceae yeast families, reproducing the industrial raisin microbiota (Fig. 1b). Based on the data described above, fresh grape-skin is likely unsuitable for *S. cerevisiae*’s stable residence, but once *S. cerevisiae* cells are exogenously added to the ecosystem, they can effectively adapt to the grape-skin environment. To address the possible commensal behavior of *S. cerevisiae*, interaction with grape-skin resident microorganisms were further examined.

### Isolation of yeasts and related microorganisms inhabiting grape-skin

**Figure 3:**
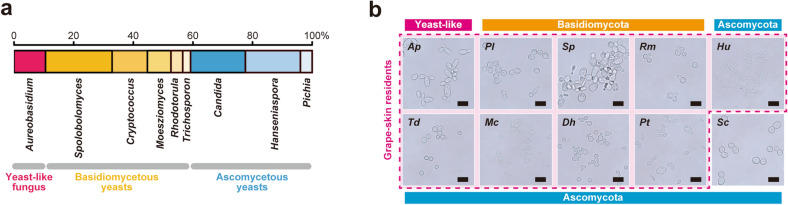
Grape-skin residents isolated in this study. (**a**) Genus-level classification of the yeasts and yeast-like microorganisms isolated in this study. “*Sporobolomyces*” includes its teleomorphic forms, *Sporidiobolus* genus. “*Cryptococcus*” includes *Papiliotrema* and *Tremella* genera in current nomenclature. “*Candida*” includes its teleomorphic forms, *Torulaspora*, *Meyerozyma*, *Debaryomyces*, and *Zygoascus* genera. (**b**) Microscopic images of the nine species of grape-skin residents and *S. cerevisiae* X2180 (*Sc*). Grape-skin residents include one yeast-like fungus *A. pullulans* (*Ap*), three basidiomycetous yeasts, *P. laurentii* (*Pl*), *S. pararoseus* (*Sp*), and *R. mucilaginosa* (*Rm*), and five ascomycetous yeasts, *H. uvarum* (*Hu*), *T. delbrueckii* (*Td*), *M. caribbica* (*Mc*), *D. hansenii* (*Dh*), and *P. terricola* (*Pt*). Bars = 10 μm.

Among chloramphenicol-tolerant, yeast-like colonies isolated from grape juice, surface-washed suspensions, or enrichment cultures, 76 clones were identified to species level by sequencing the ITS region of the nuclear rRNA gene (Supplementary Table S1). Isolated microorganisms were classified as yeast-like fungi, basidiomycetous yeasts, or ascomycetous yeasts (Fig. 3a). The yeast-like fungi included a single ascomycetous species *A. pullulans*^33–35^, also known as “black yeast”, frequently isolated from grape and wine environments in previous reports^3,4,6,8^. The isolated basidiomycetous yeasts mainly consisted of *Sporobolomyces* (a.k.a. *Sporidiobolus* as teleomorphic forms) and *Papiliotrema* (formerly categorized as *Cryptococcus*) species^3,4,6,8^. Additionally, *Rhodotorula mucilaginosa* is a common ecological basidiomycete found in soil, air, water, and foods^34,36^. *Hanseniaspora uvarum*^34,37^ was most frequently isolated from the wine grape variety, Pinot noir, among the ascomycetous yeast species. Several other ascomycetes were assigned to *Candida* (as anamorphic status) or *Pichia* genus. In most cases, every grape sample tested in this study contained a few yeasts or associated fungal species, excluding *S. cerevisiae*. The core composition of fungal microbiota (i.e., *A. pullulans*, basidiomycetous yeasts, and ascomycetous non-*Saccharomyces* yeasts) was substantially consistent with previous studies^3,4,6,8^. Thus, grape environments may be greatly suitable for the colonization and development of non-*Saccharomyces* oligotrophic fungal microbiota. Yeast-like fungus *A. pullulans*, three basidiomycetous yeast species (*Papiliotrema laurentii*, *Sporidiobolus pararoseus*, and *R. mucilaginosa*), and five ascomycetous yeast species (*H. uvarum*, *Torulaspora delbrueckii*, *Meyerozyma caribbica*, *Debaryomyces hansenii*, and *Pichia terricola*) were used as representative grape-skin microorganisms for further comparative analysis (Fig. 3b).

### Carbon assimilation profiles of grape-skin fungi and *S. cerevisiae*

**Figure 4:**
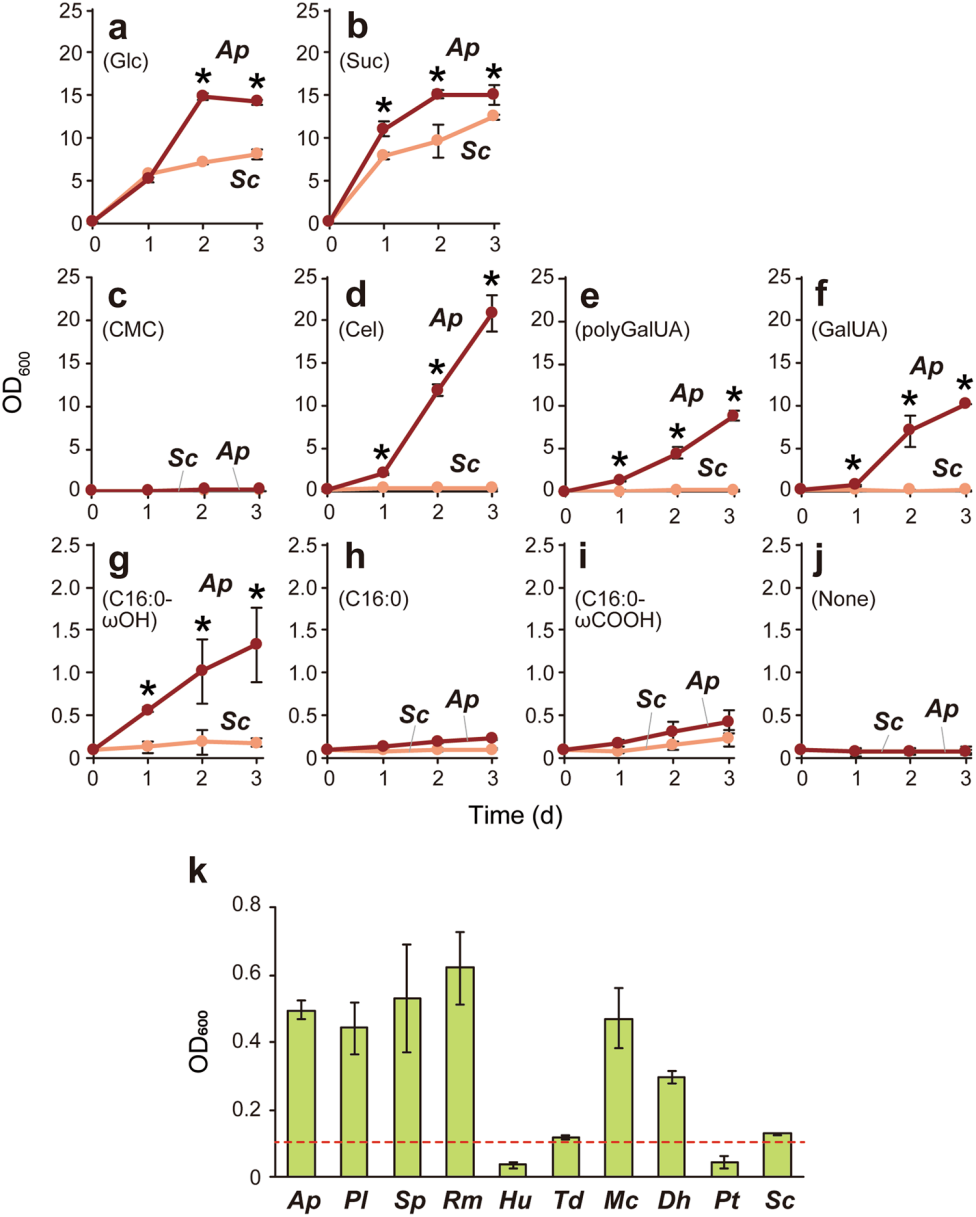
Carbon assimilation by grape-skin residents. **(a–j)** Assimilation of possible carbon sources in grape berries by *A. pullulans* and *S. cerevisiae*. Graphs indicate growth curves of *A. pullulans* (*Ap*, red) and *S. cerevisiae* X2180 (*Sc*, coral) in YNB medium containing 0.5% glucose (**a**), sucrose (**b**), CMC (**c**), cellobiose (Cel, **d**), polygalacturonic acid (polyGalUA, **e**), galacturonic acid (GalUA, **f**), ω-hydroxypalmitic acid (C16:0-ωOH, **g**), palmitic acid (C16:0, **h**), ω-carboxypalmitic acid (C16:0-ωCOOH, **i**), or no carbon source (**j**). Data represent mean values and standard deviations from three independent experiments. Asterisks indicate statistically significant increases in carbon dioxide emission compared with *Sc* (*t*-test, *p* < 0.05). (**k)** Assimilation of ω-hydroxypalmitic acid by grape-skin residents and *S. cerevisiae*. The graph indicates OD_600_ values after 24-h incubation in YNB medium containing 0.5% ω-hydroxypalmitic acid. A red dashed line shows the initial OD_600_ value (OD_600_ = 0.1). Data represent mean values and standard deviations from three independent experiments. *Ap*, *A. pullulans*; *Pl, P. laurentii*; *Sp*, *S. pararoseus*; *Rm*, *R. mucilaginosa*; *Hu*, *H. uvarum*; *Td*, *T. delbrueckii*; *Mc*, *M. caribbica*; *Dh*, *D. hansenii*; *Pt*, *P. terricola*; *Sc*, *S. cerevisiae* X2180.

To characterize carbon assimilation profile, *A. pullulans*, one of the most ubiquitously observed species among sound grape microbiota^3,4,6,8^, and *S. cerevisiae* cells were cultivated in a yeast nitrogen base (YNB) minimum medium with various possible carbon sources in grape berries (Fig. 4). Both *A. pullulans* and *S. cerevisiae* vigorously grew in the presence of fermentable sugars, such as glucose and sucrose (Fig. 4a,b). A major part of grape-skin consists of plant cell wall polymers, cellulose and pectin^17^. Although the use of carboxymethyl cellulose (CMC), a water-soluble cellulose derivative, was undetected in *A. pullulans* and *S. cerevisiae*, the main cellulose degradation product cellobiose was assimilated by *A. pullulans* (Fig. 4c,d). As previously reported^38^, the activity of carboxymethyl cellulase (i.e., CMCase) is deficient among some *A. pullulans* strains. The *A. pullulans* strain identified in this study may be unable to degrade cellulose by themselves but can grow by using cellobiose produced through cellulose degradation by other grape-skin fungi. The major pectic polysaccharide polygalacturonic acid and its building block galacturonic acid were assimilated by *A. pullulans*, but not by *S. cerevisiae* (Fig. 4e,f). Thus, *A. pullulans* likely degrades and assimilates a broad spectrum of plant cell wall-relevant materials, unmetabolized by *S. cerevisiae*, consistent with previous reports^19–22,34,35^.

The plant cuticle, the outermost hydrophobic layer, is another major component of grape-skin^17^. Cutin in the plant cuticle is a polyester of ω-hydroxy C_16_ and C_18_ fatty acids and their derivatives. We discovered that *A. pullulans* exhibited weak but reproducible growth using ω-hydroxypalmitic acid as a sole carbon source (Fig. 4g). Since neither palmitic acid or ω-carboxypalmitic acid was assimilated (Fig. 4h,i), *A. pullulans* may possess a utilization system specific for ω-hydroxy fatty acids. Besides *A. pullulans*, three basidiomycetous yeasts, *P. laurentii*, *S. pararoseus*, and *R. mucilaginosa*, and two ascomycetous yeasts, *M. caribbica* and *D. hansenii*, showed significant growth in the presence of ω-hydroxypalmitic acid (Fig. 4k).

### Cutinase-like esterase activity in *A. pullulans*

**Figure 5:**
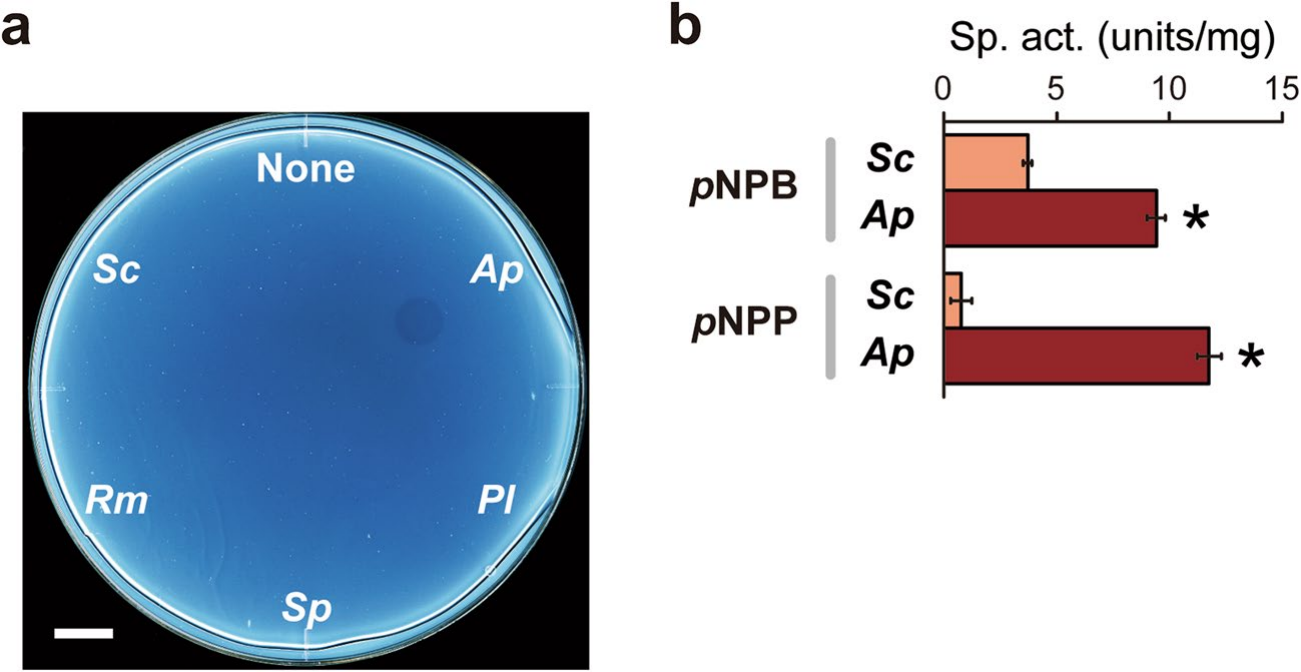
Cutinase-like activity of the culture supernatants of grape-skin residents and *S. cerevisiae*.** (a)** PCL-plate clearing assay. (**b)** *p*NPB and *p*NPP hydrolysis assay. The supernatants were obtained from fully grown 5-d cultures in a YNB medium containing 2% (w/v) glucose. Data represent mean values and standard deviations from three independent experiments. *Ap*, *A. pullulans*; *Pl, P. laurentii*; *Sp*, *S. pararoseus*; *Rm*, *R. mucilaginosa*; *Sc*, *S. cerevisiae* X2180. Asterisks indicate statistically higher specific activity than *Sc* (*t*-test, *p* < 0.05).

The secretion of cutin-degrading enzymes in *A. pullulans*, basidiomycetous yeasts, and *S. cerevisiae* was tested using a model polyester polycaprolactone (PCL)-plate (Fig. 5a). Known cutinases from the other species represent PCL degradation activity in previous studies^39–41^. The supernatant of the fully grown *A. pullulans* culture in YNB medium plus 2% glucose formed a clear halo, although no halo was observed using the other supernatant samples. Additionally, the supernatant of the *A. pullulans* culture exhibited higher esterase activity toward *p*-nitrophenyl butyrate (*p*NPB) and *p*-nitrophenyl palmitate (*p*NPP) used as substrates than the supernatant of the *S. cerevisiae* culture (Fig. 5b). These data suggest that the isolated *A. pullulans* strain from grapes secretes cutinase-like esterase to assist penetration into plant cuticles.

**Figure 6:**
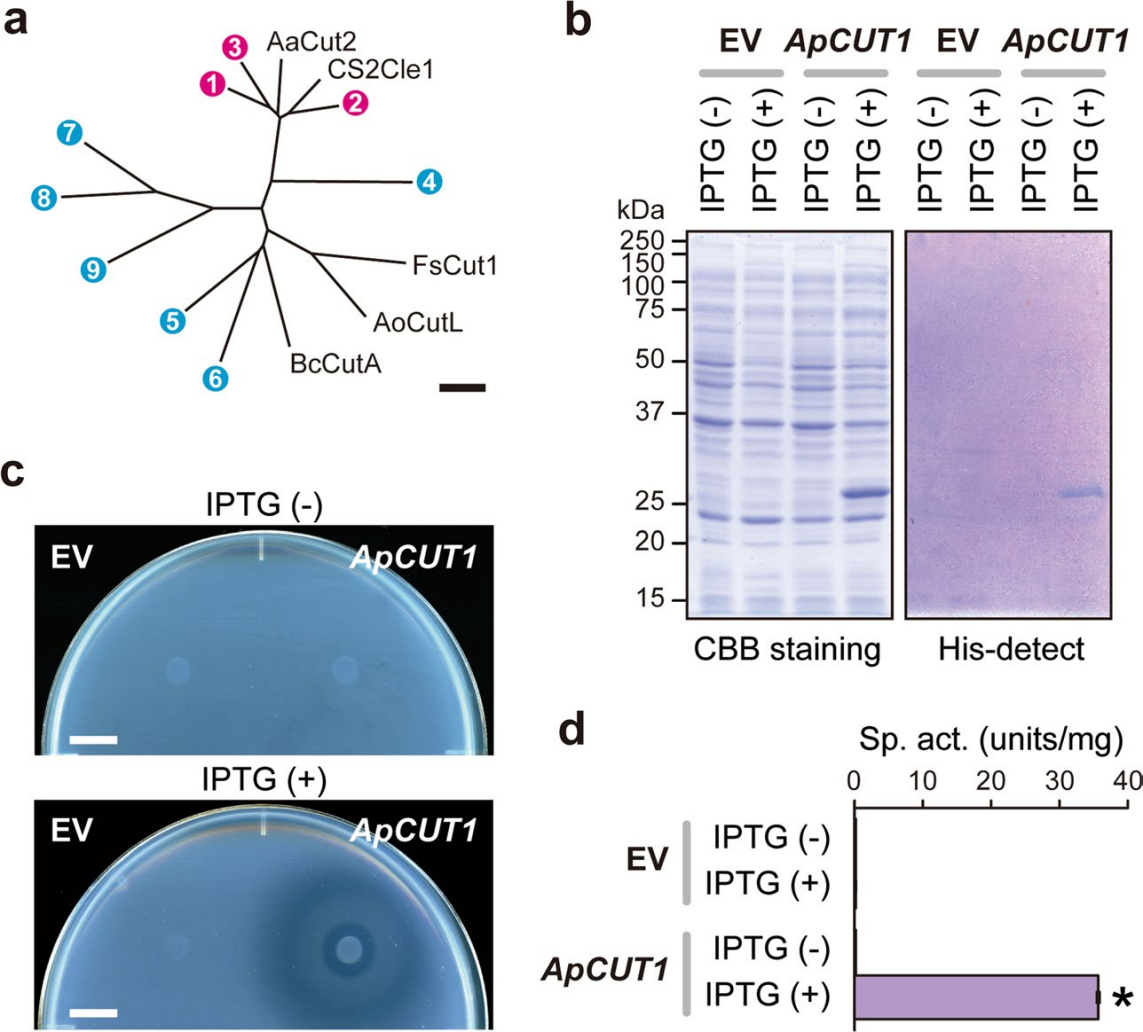
Cutinase-like activity of recombinant ApCut1**. (a)** A phylogenetic tree of *A. pullulans* cutinase-like gene products. The number corresponds to each cutinase-like gene product in *A. pullulans*. ApCut1–ApCut3 (magenta) belongs to the yeast cutinase family, while ApCut5 to ApCut9 (cyan) belong to the mold cutinase family. Bar, 0.2 substitutions per nucleotide position. (**b)** Expression of recombinant ApCut1 in *E. coli*. Left and right panels indicate CBB-stained gel and His-detect-stained gel for specific detection of His-tagged proteins, respectively. (**c)** PCL-plate clearing assay. Bar, 1 cm. (**d)** *p*NPB hydrolysis assay. Data represent mean values and standard deviations from three independent experiments. An asterisk indicates a statistically higher specific activity than the negative control (IPTG (-), *t*-test, *p* < 0.05). EV, empty vector.

Previous whole-genome analysis of the *A. pullulans* EXF-150 strain^42^ revealed nine candidate genes encoding cutinase-like enzymes, designated as ApCut1 to ApCut9 (Supplementary Fig. S1). These gene products contained a classical α/β-hydrolase catalytic triad Ser-His-Asp and a Gly-Tyr-Ser-Gln-Gly (GYSQG) motif conserved among cutinase catalytic sites^29,35^. Additionally, two pairs of cysteine residues forming disulfide bonds, important for spatial conformation^29,43^, were also conserved in all cutinase candidates except for ApCut4, in which the amino terminus is truncated. Phylogenetic analysis indicated that ApCut1 to ApCut3 form a subgroup with yeast cutinases from *Arxula adeninivorans* and *Cryptococcus* sp. S-2^39,41^, while ApCut5 to ApCut9 form a subgroup with mold cutinases from *Fusarium solani*, *Aspergillus oryzae*, and *Botrytis cinerea* (Fig. 6a)^44–46^. Since the yeast-type cutinases were less characterized than mold cutinases so far, we decided to overexpress ApCut1 to ApCut3 to test their enzymatic activity. Whole-cell lysate of *E. coli* expressing recombinant ApCut1 indicated cutinase-like activity, based on the PCL-plate clearing assay and the *p*NPB hydrolysis assay (Fig. 6b–d). Also, the extracts of ApCut2- or ApCut3-expressing *E. coli* cells weakly degraded PCL (Supplementary Fig. S2). Since the expression of ApCut2 and ApCut3 was almost undetectable in the coomassie brilliant blue (CBB)-stained gel, more attention should be paid to the protease sensitivity, expression conditions, and synonymous codon usage bias. These results revealed the activity of the yeast-type cutinase isoenzymes in *A. pullulans*. Which and how *A. pullulans* genes are expressed under plant cuticles environments are major issues to be solved.

### Alcoholic fermentation of glucose by grape-skin fungi and *S. cerevisiae*

**Figure 7:**
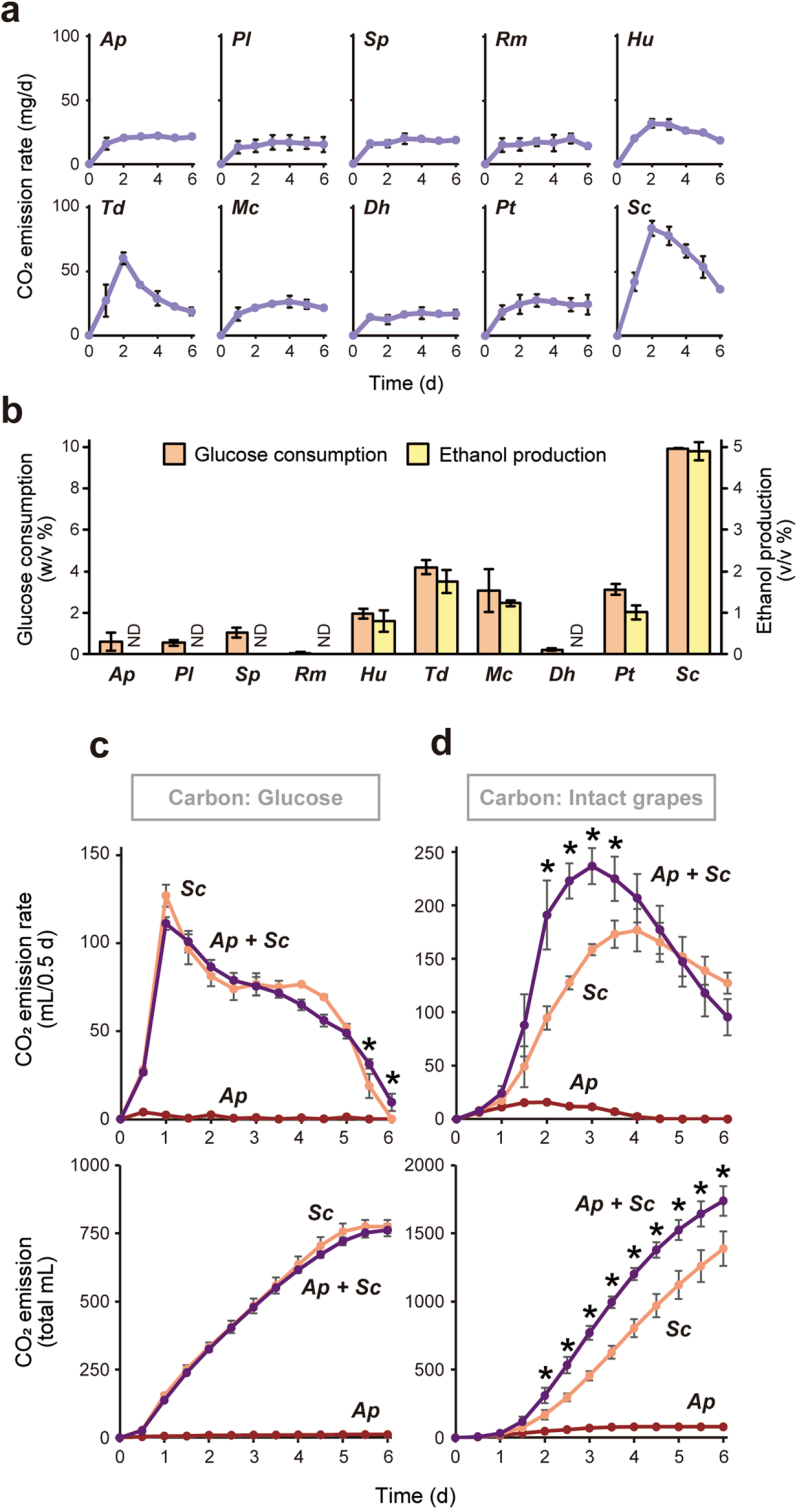
Alcoholic fermentation by grape-skin residents. **(a,b)** Alcoholic fermentation of glucose by grape-skin residents and *S. cerevisiae*. Carbon dioxide emission rate during 6-d alcoholic fermentation in YNB medium containing 10% (w/v) glucose (**a**). Glucose consumption (orange) and ethanol production (yellow) during 6-d alcoholic fermentation in YNB medium containing 10% (w/v) glucose (**b**). Data represent mean values and standard deviations from three independent experiments. *Ap*, *A. pullulans*; *Pl, P. laurentii*; *Sp*, *S. pararoseus*; *Rm*, *R. mucilaginosa*; *Hu*, *H. uvarum*; *Td*, *T. delbrueckii*; *Mc*, *M. caribbica*; *Dh*, *D. hansenii*; *Pt*, *P. terricola*; *Sc*, *S. cerevisiae* X2180. (**c,d)** Alcoholic fermentation of glucose or intact grapes in coculture of* A. pullulans* and *S. cerevisiae*. Carbon dioxide emission rate (upper) and total carbon dioxide emission (lower) during 6-d alcoholic fermentation in YNB medium containing 10% (w/v) glucose (**c**) or in a mixture of an equal weight of YNB medium and intact grape berries (**d**). Data represent mean values and standard deviations from three independent experiments. *Ap*, inoculated with *A. pullulans* (red); *Sc*, inoculated with *S. cerevisiae* X2180 (coral); *Ap* + *Sc*, coinoculated with *A. pullulans* and *S. cerevisiae* X2180 (violet). Asterisks indicate statistically significant increases in carbon dioxide emission compared with *Sc* (*t*-test, *p* < 0.05).

To assess alcoholic fermentation performance of the grape-skin fungi and *S. cerevisiae*, each microorganism was statically incubated in a 5-mL liquid YNB medium, synthetic minimum medium for laboratory yeast strains, supplemented with 10% (w/v) glucose as a sole carbon source. As all species grew well in this medium (Supplementary Fig. S3; optical density of 600 nm (OD_600_) > 1), carbon dioxide emission rate, glucose consumption, and ethanol production were quantified during the 6-d fermentation test (Fig. 7a,b). *S. cerevisiae* exhibited a robust peak of fermentation rate and full glucose consumption to yield approximately 5% (v/v) ethanol. In contrast, yeast-like fungus *A. pullulans*, basidiomycetous yeasts, *P. laurentii*, *S. pararoseus*, *R. mucilaginosa*, and ascomycetous yeast *D. hansenii* showed a constant basal level of carbon dioxide emission, no detectable ethanol production, and little glucose consumption, and was categorized as non-fermenting species. The other ascomycetous yeasts, *H. uvarum*, *T. delbrueckii*, *M. caribbica*, and *P. terricola*, with intermediate phenotypes, were categorized as weak-fermenting species.

We mainly focused on the coculture effect of non-fermenting *A. pullulans* on the alcoholic fermentation ability of *S. cerevisiae*. Under the coculture condition of *S. cerevisiae* and non-fermenting species, alcoholic fermentation is solely attributed to *S. cerevisiae*. In the coculture experiment in 50-mL YNB medium containing 10% (w/v) glucose as a sole carbon source, the non-fermenting species *A. pullulans* displayed at least no significant interaction with *S. cerevisiae* that could be detected by carbon dioxide emission in alcoholic fermentation (Fig. 7c). The coculture of *A. pullulans* and *S. cerevisiae* may have no synergistic effect on the growth of both species (Supplementary Fig. S4). We note that the coculture exhibited a pink color, suggesting growth of *A. pullulans* (red) and *S. cerevisiae* (white) strains.

### Alcoholic fermentation of intact grapes by nonfermentative, grape-skin fungi and *S. cerevisiae*

In 50-mL YNB minimum medium plus 50-g intact grape berries as a sole carbon source (Fig. 7d), *A. pullulans* generated almost no carbon dioxide, whereas *S. cerevisiae* slowly progressed alcoholic fermentation, reaching the maximum carbon dioxide emission rate 4 d after inoculation. Since robust peaks of fermentation rates were typically observed within 1−2 d after inoculation in the presence of glucose (Fig. 7a,c), grape-skin may function as a physical barrier against *S. cerevisiae* cells to protect fermentable sugars, such as glucose, fructose, and sucrose, inside grape berries.

When *A. pullulans* and *S. cerevisiae* were co-cultured, the initial carbon dioxide emission rate increased, and a maximum fermentation rate was observed at 3 d (Fig. 7d). Considering the minor contribution of *A. pullulans* to alcoholic fermentation and *S. cerevisiae* growth, *A. pullulans* may specifically contribute to an increase in sugar availability for *S. cerevisiae*, leading to accelerated alcoholic fermentation. Coculture of *S. cerevisiae* with the nonfermentative basidiomycetous yeast, *P. laurentii*, *S. pararoseus*, or *R. mucilaginosa*, gave similar results (Supplementary Fig. S5). A possible hypothesis accounting for these data is that *S. cerevisiae* cells might make use of the ability of grape-skin microbiota to access fermentable sugars in grape berries upon triggering spontaneous wine fermentation.

## Discussion

Based on the experimental data, we propose that the grape-skin-resident microorganisms, including nonfermentative, yeast-like fungus *A. pullulans*, increase the accessibility to fermentable sugars in intact grape berries by degrading and assimilating the plant cell wall, cuticle compounds, or both. Grape-skin microbiota’s high and versatile abilities to degrade and assimilate plant cell wall and/or cuticle is likely to be essential for their adaptation and proliferation on grape-skin. In contrast, *S. cerevisiae* cells without such abilities need the aid of grape-skin microbiota to survive in grape environments. Studies of wild populations of *S. cerevisiae* and its closest relative *Saccharomyces paradoxus* suggest that woodlands or primeval forests are natural habitats for these yeast species^47–49^. In this study, there was no *S. cerevisiae* clone isolated even from fermented juice or enriched cultures in 5% sucrose. Moreover, *S. cerevisiae* was unable to assimilate plant cell wall, cuticle, and their components. Since our original motivation was to search for wine yeasts responsible for spontaneous alcoholic fermentation, no bacterial screening was performed in this study. In fact, our finding supported that wine yeasts are unlikely derived from grape-skin, consistent with previous reports^2–4^. Altogether, nutrient-poor, intact grape surfaces may be inappropriate for wine yeasts as their stable and permanent habitats. Although *Saccharomyces* species might have been accidentally brought to the vineyard by wind or yeast-carrier animals^10,11^, the incidence of *S. cerevisiae* wine yeasts still needs to be experimentally explored. Our results provide an important clue to address how *S. cerevisiae* cells met and conquered grapes upon the origin of spontaneous wine fermentation.

What are the key grape-skin compounds that protect fermentable sugars in intact grape berries? Based on the assimilation tests of CMC and cellobiose (Fig. 4c,d), our isolated *A. pullulans* strain lacks cellulase. This is consistent with a previous report about intraspecific variations of cell wall-degrading enzymes in *A. pullulans*^38^. Thus, cellulose degradation may be nonessential for accelerated alcoholic fermentation of intact grapes. Among pectin-degrading enzymes, polygalacturonase is genetically encoded and expressed by grape-skin resident species and by *S. cerevisiae*^50,51^. Recently, we revealed the importance of pectin as an initial target for the saprophytic bacterium *Bacillus subtilis* to recognize the surface of dead soybeans^52^, whereas it is unlikely that pectin degradation by non-*Saccharomyces* microorganisms specifically accelerated alcoholic fermentation of intact grapes. As shown in Fig. 5a, the PCL-degrading activity was detected in *A. pullulans*, although not in the examined basidiomycetous yeast strains. Based on these data, the responsible enzymatic activity specific and common to all grape-skin residents is still unidentified. The degradation of the other plant cell wall or cuticle components should be focused on in future research. Furthermore, cooperative and synergistic degradation by whole grape-skin microbiota, including not only fungi but also bacteria, needs to be investigated. It is also noted that grapes and raisins used in this study are commercial and there is no way to know the influence of pre- and post-harvest treatments. We should start considering the impact of typical grapevine pathogenic microorganisms^53^, such as *B. cinerea*, with strong degradative capacity to invade into plant bodies, on the interaction with *S. cerevisiae* upon the origin of wine. Such complicated, highly ordered microbial interactions at the chemical, metabolic, genetic, and genomic levels will be the central issue in applied microbiology and microbial ecology.

This is the first report on ω-hydroxypalmitic acid assimilation as a carbon source by *A. pullulans* and several yeast species. Although ω-hydroxylation of fatty acids also occurs in mammals and insects, ω-hydroxy fatty acids play a broad and vital biological role in higher plants as major components of cutin and suberin^25–27,54–56^. Thus, the microbial ability to assimilate ω-hydroxy fatty acids may mainly contribute to symbiotic interactions with terrestrial plants. In the ω-oxidation process of animals and plants, known as a minor, fatty acid catabolic pathway, a hydroxy group is introduced onto the ω carbon of the medium to long-chain fatty acids^55,56^. The resultant ω-hydroxy fatty acids are oxidized to ω-carboxy fatty acids (i.e., dicarboxylic fatty acids), further degraded through the β-oxidation pathway. Grape-skin residents may probably metabolize ω-hydroxy fatty acids similarly. Based on our data, *A. pullulans* cells are suggested to have an ω-hydroxy fatty acid-specific transporter because they can grow using ω-hydroxypalmitic acid as a sole carbon source, not palmitic acid or ω-carboxypalmitic acid. Furthermore, this study revealed that *A. pullulans* may secrete cutinase to hydrolyze cutin into ω-hydroxy fatty acids and other minor components, such as glycerol. Both aspects of cutin degradation and ω-hydroxy fatty acid assimilation may characterize *A. pullulans* as the most abundant and persistent resident among grape-skin microbiota. Notably, the *A. pullulans* EXF-150 strain was first identified as a microorganism that acquired both yeast- and mold-type cutinase genes in the genome, which may be associated with the yeast-to-hyphal dimorphic transition of this species^33^. Enzymatic analysis of yeast- and mold-type cutinases in *A. pullulans* will reveal the significance of differences between both cutinase types.

Our analysis of microbiota in raisins and raisin yeast provided important clues to the archetype of natural wine fermentation. Since dried raisins were found to contain a conciderable amount of *S. cerevisiae* or its relatives, a decrease in sugar concentration by mixing with water enables yeast cells to avoid high osmotic stress and induce vigorous alcoholic fermentation. As a result, ethanol-tolerant microorganisms, such as *S. cerevisiae*, govern the population in raisin water (Fig. 1). Although *S. cerevisiae* was not found on aseptically dried grape berries, we here presented the evidence that inoculated *S. cerevisiae* can grow on the surface of grape berries (Fig. 2). Thus, colonist *S. cerevisiae* may adapt to the grape-skin environments and conquer other microorganisms in response to the optimal sugar concentration for yeast growth and alcoholic fermentation. As grape-skin is unlikely the original habitat for *S. cerevisiae*, the initial adaptation step may be the key to understand the origin of wine. Now we are further addressing how *S. cerevisiae* copes and interacts with grape-skin habitants to survive and establish the growth on the grape environment, based on metabolite dynamics.

**Figure 8:**
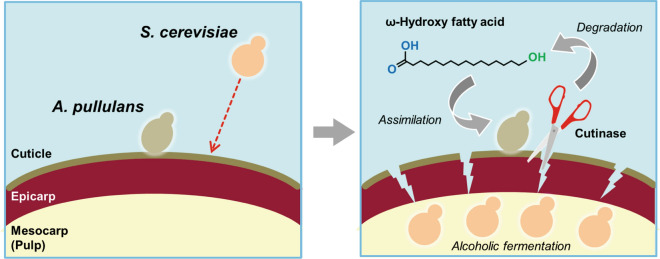
Tripartite interaction model between a grape berry, grape-resident *A. pullulans*, and wine yeast *S. cerevisiae* from the perspective of winemaking origin.

Conclusively, this study focused on the symbiosis between grape-skin microbiota and *S. cerevisiae* from the perspective of winemaking origin (Fig. 8). Oligotrophic microorganisms, such as *A. pullulans*, have developed versatile abilities to use plant cell wall polysaccharides and plant cuticular lipids as nutrient sources to establish their ecological niche. Especially, degradation and assimilation of the plant cuticle, the outermost layer interacting with the environment, may be a prerequisite for oligotrophic resident microorganisms to trigger colonization and adaptation. In contrast, eutrophic yeasts, including *S. cerevisiae*, yield energy through the alcoholic fermentation of sugars in grape berries with the aid of oligotrophic microorganisms. Such tripartite interaction between grape berries, oligotrophic residents, and eutrophic yeasts determines the grape-skin microbiome dynamics and spontaneous wine fermentation. Moreover, since grape bacterial microbiota^7–9^ remain elusive in this study, the individual interactions between bacteria and grape berries, between bacteria and oligotrophic eukaryotic residents, or between bacteria and eutrophic yeasts should also be investigated in future. Thus, studying the origins and microbiota ecological succession in fermented foods will help elucidate the key principles governing plant-microbial ecosystems’ emergence and development.

## Methods

### ITS amplicon sequence analysis of raisin microbiota

To prepare raisin yeast, California Green Seedless raisins, non-oil coated, from different manufacturers were purchased from Tomizawa Shouten (Japan), Imagawa Seika (Japan), Nishiuchi Kagetsudo (Japan), and Fujimoto Shouten (Japan). Raisin microbiota were prepared from surface-washed suspensions. 100-g raisins and 300-mL sterilized water were mixed and incubated at 30 °C for 14 d. For the raisin making experiment, approximately 300-g (per batch) fresh grape berries of Green Seedless were dried in an forced air flow oven (WFO-520W, Eyela, Japan) at 37 °C for 21 d. *S. cerevisiae* X2180 cells, obtained from the American Type Culture Collection (USA), were fully grown in the rich medium, washed by sterilized water, and inoculated on the surface of individual grape berries at the cell density of 10 cells/berry. X2180 was also used for other experiments as a representative of diploid *S. cerevisiae* strains (*Sc*) in this study.

Both DNA extraction and ITS amplicon sequence analysis were performed by Bioengineering Lab. Co., Ltd. (Sagamihara, Japan) based on a previously reported method^57^. In summary, the ITS1 region was amplified using the ITS1F_KYO1/ITS2-KYO2 primer sets. Sequencing was conducted using a paired-end, 2 × 300 bp cycle run on a MiSeq sequencing system (Illumina) and MiSeq Reagent Kit version 3 chemistry. After quality filtering and chimera check of sequencing reads, the operational taxonomic units (OTU) were predicted using QIIME 2^58^. The OTUs that accounted for more than 1% of the total sequence number were classified, whereas taxa with abundance < 1% were summarized as “others”.

### Isolation and identification of grape-skin microorganisms

To isolate yeasts or yeast-like fungi, (i) juice, (ii) surface-washed suspensions, or (iii) enrichment cultures were obtained from commercially available wine or table grape varieties belonging to *Vitis vinifera* species or *Vitis* interspecific hybrids (see Supplementary Table S1). The use of plants in the present study complies with the IUCN Policy Statement on Research Involving Species at Risk of Extinction and the Convention on the Trade in Endangered Species of Wild Fauna and Flora. Grape juice was obtained from freshly pressed grape berries using a food-grade juicer (Panasonic, Japan) and was used either immediately or after being incubated at 30 °C for 3 d. Surface-washed suspensions were obtained by vigorously shaking the flask containing approximately 50-g grape berries and 25-mL sterilized water at 30 °C for 15 min^59^. Note that the washing did not destroy the sound grape berries used. Enrichment cultures were obtained by statically incubating the flask containing approximately 10-g grape berries and 40-mL sterilized water or 5% (w/v) sucrose at 30 °C for 3 d. Each sample was spread on a nutrient-rich,YPD (1% yeast extract, 2% peptone, and 2% glucose) medium plate with 0.1% chloramphenicol to inhibit bacterial growth, and was incubated at 30 °C. Single colonies representing yeast-like colony morphology were isolated by repeatedly streaking on YPD medium plates with 0.1% chloramphenicol.

The isolated clones were identified by DNA sequencing of the rRNA gene ITS region^60^, using the ITS_1F (5′-GTAACAAGGTYTCCGT-3′) and ITS_1R (5′-CGTTCTTCATCGATG-3′) primer pair and genomic DNA as PCR templates. Genomic DNA was extracted and purified using the Dr. GenTLE (from Yeast) High Recovery (Takara Bio, Japan). Each 25 µL PCR reaction consisted of 1 µL of approximately 100 ng/µL template DNA, 12.5 µL of 2× PCR buffer for KOD FX Neo, 0.5 µL of KOD FX Neo (Toyobo, Japan), 10 µM forward primer, 10 µM reverse primer, and 10 mM dNTPs. The PCR conditions were: 94 °C for 2 min for initial denaturing, then 98 °C for 10 s, 55 °C for 30 s, and 68 °C for 30 s for 30 cycles, then 16 °C. The products were purified using Gel/PCR DNA isolation system (Viogene, USA). DNA sequencing was performed by Macrogen Japan (Japan), using both the ITS_1F and ITS_1R primers. Species were identified by searching databases using the BLAST sequence analysis tool (http://www.ncbi.nlm.nih.gov/BLAST/). Since it was difficult to get a clean reading of the total length of the ITS for all isolated strains, these analyses should be considered at the preliminary level. In the nine representative grape-skin resident species (*Ap*, *Pl*, *Sp*, *Rm*, *Hu*, *Td*, *Mc*, *Dh*, and *Pt*) used in the later experiments, the sequence of the D1/D2 region of 26S rDNA was determined by TechnoSuruga Laboratory (Japan).

### Carbon assimilation test

Cells were aerobically precultured at 30 °C for 2 d in YNB minimum medium containing 2% (w/v) glucose as a carbon source, harvested, washed by sterilized water, and inoculated into 5 mL of YNB medium containing 0.5% (w/v) of carbon sources as below: glucose, sucrose, CMC, cellobiose, polygalacturonic acid, galacturonic acid, palmitic acid, 16-hydroxyhexadecanoic acid (i.e., ω-hydroxypalmitic acid), or heptadecanedioic acid (i.e., ω-carboxypalmitic acid). Test tubes were aerobically shaken at 130 strokes per minute at 30 °C for 3 d. Upon inoculation, initial OD_600_ was adjusted to 0.1. For assimilation tests of palmitic acid, ω-hydroxypalmitic acid, or ω-carboxypalmitic acid, 0.05% (w/v) Tween 40 was added to the medium. In advance of measurement of OD_600_, insoluble fatty acids were removed by washing pellets with hexane three times, and cells were dissolved in sterilized water^61^. All growth tests were repeated at least three times. Data were statistically analyzed with the two-tailed Student's *t*-test.

### Phylogenetic analysis of yeast and mold cutinases

Amino acid sequences of nine putative cutinase gene products in the *A. pullulans* EXF-150 strain and well-studied yeast and mold cutinases [Cut2 from *Arxula adeninivorans* (AaCut2)^41^, Cle1 from *Cryptococcus* sp. strain S-2 (CS2Cle1)^39^, Cut1 from *Fusarium solani* (FsCut1)^40^, CutL from *Aspergillus oryzae* (AoCutL)^45^, and CutA from *Botrytis cinerea* (BcCutA)^46^] were obtained from UniPlotKB. Multiple sequence alignment was conducted using Clustal Omega program, and the phylogenetic tree was constructed using Molecular Evolutionary Genetics Analysis v.10.2.2. The putative cutinase genes of *A. pullulans* were designated as below: M438DRAFT_340638 as *ApCUT1*, M438DRAFT_351543 as *ApCUT2*, M438DRAFT_352218 as *ApCUT3*, M438DRAFT_388226 as *ApCUT4*, M438DRAFT_264999 as *ApCUT5*, M438DRAFT_267580 as *ApCUT6*, M438DRAFT_341517 as *ApCUT7*, M438DRAFT_347465 as *ApCUT8*, and M438DRAFT_368700 as *ApCUT9*.

### Expression of recombinant *A. pullulans* cutinase

The chemically synthesized *ApCUT1* gene (Eurofins, Luxembourg) was cloned into the BamHI-EcoRI site of the pET-21b(+) vector (Merck Millipore, USA) to express recombinant ApCut1p tagged with the T7 epitope at the amino terminus and the 6× His epitope at the carboxy terminus. The resultant pET-21b(+)-*ApCUT1* plasmid was introduced into *Escherichia coli* BL21(DE3)pLysS cells (Novagen, Germany). A transformant was inoculated into 100-mL Luria–Bertani medium (0.5% yeast extract, 1% tryptone, and 1% sodium chloride) with 100-μg/mL ampicillin and 34-μg/mL chloramphenicol, and was cultured at 37 °C to an OD_600_ of 0.6. Isopropyl thio-β-d-galactoside (IPTG) was added to the culture to a final concentration of 1 mM, and cells were further cultured at 37 °C for 4 h. Whole-cell extracts were prepared from cell pellets suspended in the xTractor buffer (TakaraBio, Japan), followed by the addition of DNase I. After protein separation by SDS-PAGE, the proteins were detected by CBB staining or using His-Detect In-Gel Stain (Nacalai Tesque, Japan). Full-length gels are displayed in Supplementary Fig. S6.

### Enzymatic assay

To assay the potential cutinase activity from the culture supernatants of fully grown *A. pullulans* or yeasts, PCL was used as a model polyester substrate. Turbid agar plates containing 0.05% PCL (Fujifilm Wako Pure Chemical, Japan) were prepared as previously described^40,41^. The assay for determining esterase activity was conducted according to previous reports^41,62^, using *p*NPB and *p*NPP (Sigma-Aldrich, USA) as substrates. All assays were repeated at least three times.

### Fermentation test

Cells were aerobically precultured at 30 °C for 2 d in 0.67% YNB minimum medium containing 2% (w/v) glucose as a carbon source and harvested. For the alcoholic fermentation of glucose, cells were inoculated into a YNB medium containing 10% (w/v) glucose at a final OD_600_ of 0.1 and were then further incubated at 30 °C without shaking. For the alcoholic fermentation of intact grapes, cells were inoculated into 50-mL YNB medium at a final OD_600_ of 0.1, mixed with approximately 50-g commercially available intact grape berries of Green Seedless, and were then further incubated at 30 °C without shaking. Fermentation was continuously monitored by measuring the weight loss of evolved carbon dioxide for 5-mL test tube-scale tests or using a Fermograph II apparatus (Atto, Japan) for 50-mL-scale coculture tests. Glucose and ethanol concentrations were determined using the LabAssay glucose kit (Fujifilm Wako Pure Chemical, Japan) and the ethanol assay F-kit (Roche, Switzerland), respectively. All fermentation tests were repeated three times. Data were statistically analyzed with the two-tailed Student's *t*-test.

## Supplementary Information

Supplementary Information.

## Data Availability

All sequences determined in the ITS amplicon sequence analysis using raisin yeast and dried grape berry samples have been deposited in the DNA Data Bank of Japan (DDBJ) under the accession numbers DRA015192 and DRA015183, respectively. All sequences of the D1/D2 region of 26S rDNA in the representative grape-skin resident species have been deposited in the DDBJ under the accession numbers, LC765834-LC765842.
