# Supplementary material for: Adaptation of yeast Saccharomyces cerevisiae to grape-skin environment
Source: Sci Rep. 2023 Jun 20;13:9279. doi: 10.1038/s41598-023-35734-z (PMC10281991; doi:10.1038/s41598-023-35734-z)
Supplement: Supplementary file 1 — Supplementary Information. [file 41598_2023_35734_MOESM1_ESM.pdf]

Supplementary Information

Adaptation of yeast *Saccharomyces cerevisiae* to grape-skin environment

Daisuke Watanabe[1,2] and Wataru Hashimoto[1,*]

[1]Laboratory of Basic and Applied Molecular Biotechnology, Division of Food Science and Biotechnology, Graduate School of Agriculture, Kyoto University, Uji, Kyoto, Japan
[2]Laboratory of Applied Stress Microbiology, Division of Biological Science, Graduate School of Science and Technology, Nara Institute of Science and Technology, Ikoma, Nara, Japan

Correspondence and request for materials should be addressed to W.H. (e-mail: hashimoto.wataru.8c@kyoto-u.ac.jp).

Supplementary Fig. S1 Alignment of amino acid sequences of known cutinases and ApCut1 to ApCut9.
Supplementary Fig. S2 PCL-plate clearing assay of the extracts of ApCut1- to ApCut3-expressing *E. coli* cells.
Supplementary Fig. S3 Growth of grape-skin residents and *S. cerevisiae* in YNB medium containing 10% (w/v) glucose as a sole carbon source.
Supplementary Fig. S4 Growth in coculture of *A. pullulans* and *S. cerevisiae* in YNB medium containing 10% glucose as a sole carbon source.
Supplementary Fig. S5 Alcoholic fermentation of intact grapes in coculture of grape-skin residents and *S. cerevisiae*.
Supplementary Fig. S6 Full-length gel images of Fig. 8b.
Supplementary Table S1 Yeasts or yeast-like microorganisms isolated from grapes in this study.

Supplementary Fig. S1

```
ApCut9          ------------------------------------------------------------   0
ApCut8          MSPRSVTLLSALLAASSVSAAPAALQKRDMVTAVIDGQTVSWENNWFGAPAPTAAPTAIP   60
ApCut7          ------------------------------------------------------------   0
ApCut4          ------------------------------------------------------------   0
AaCut2          ------------------------------------------------------------   0
CS2Cle1         ------------------------------------------------------------   0
ApCut1          ------------------------------------------------------------   0
ApCut3          ------------------------------------------------------------   0
ApCut2          ------------------------------------------------------------   0
FsCut1          ------------------------------------------------------------   0
AoCutL          ------------------------------------------------------------   0
ApCut6          ------------------------------------------------------------   0
ApCut5          ------------------------------------------------------------   0
BcCutA          ------------------------------------------------------------   0

ApCut9          ---------------------------------------MKSAIAAIPLLLV--------   13
ApCut8          SSVAAAASSSAAANNAGGQWITASFAGEVFSWQVNAPTATPAATSAAPAPAQ--------   112
ApCut7          -------------------------------MRVAAPSLAL--------ALA--------   13
ApCut4          ------------------------------------------------------------   0
AaCut2          ----------------M------------K----ANLILACASL----------------   12
CS2Cle1         ---------------ML------------V----SALALA--VLSAASLGRA----A---   20
ApCut1          ---------------MH------------F----TNSILAGAS----ILG----------   15
ApCut3          ----------------M------------F----SDISLKGALLSVFLAA----------   18
ApCut2          ----------------M------------S----SILAWTKAACLLLLAS----------   18
FsCut1          ---------------MK------------F---FALTTLLAATASALPTSNPAQE----L   26
AoCutL          ---------------MH------------L---RNIVIALAATAVASPV-----D----L   21
ApCut6          ---------------MGG--LGSSSTGSSD---SSASTGTATTD--SSSGSSGFGSLGSL   38
ApCut5          ---------------MK-----SF-----F---IACLAATSTLA--APLAVPQFGGFPSF   30
BcCutA          ---------------MK-----TS-----A---Q---QLLSALL--LPLSV---------   18

ApCut9          -----GSSMAA------PLEKRACPNIHIFGARETTAPAGYGSA-GT-VVNLILN--AHP   58
ApCut8          -----PTSVAAPQPSQAGNTDPSCKDVHIFLSKGWNEPY-PGRQ-GK-LAGAICY--GLD   162
ApCut7          -----QTSVAA-TVPQLEARQSACSDVHIFLAKGNNEPY-PGRQ-GK-LVNAICD--GLD   62
ApCut4          ------------------------------------------MMFQT-LVSRVLQN-TTG   16
AaCut2          --------VAGVSAAPLERRDGGCSKYTIIDTRGTGELQGPSAGFIT-MNRNILSQ-VPG   62
CS2Cle1         ----PTPESAEAHELEARATSSACPQYVLINTRGTGEPQGQSAGFRT-MNSQITAA-LSG   74
ApCut1          --------LAAASPIELDTRQSGCSSYTIINTRGTGEAQGPSAGFRT-MNSNILSS-KSG   65
ApCut3          --------GAVASP--IEKR-AACSQYTIISTRGTGELQGPSAGFRT-MNQQTLAQ-VSG   65
ApCut2          --------EAFASPIELEKR-AVCTAYTLIETRGTGEPQGPSNSFKT-MDAAIIAQ-LAG   67
FsCut1          E---ARQLGRTTRDDLINGNSASCRDVIFIYARGSTETGNLGTL-GPSIASNLESAFGKD   82
AoCutL          Q---DRQL--TGGDEL---RDGPCKPITFIFARASTEPGLLGIS-TGPAVCNRLKLARSG   72
ApCut6          GSFTGSAGGAVPSNDV--TSNTGCKKVTFIFARGTTEMGTLGSVVGPGLAQDLIKDTGS-   95
ApCut5          GGGFGGGFGGSTKNDV--T-SGVCKPVTYIFARGTTELGNMGSTVGPALEKALESAFGAN   87
BcCutA          --------LAAPTGSI--E-ARACSDVTVIFARGTTETGTLGTVVGPPFLAALKSALGSS   67

ApCut9          GSTAE--VINYPAAGGDSYASS----VQAGVKAVTNQINSFAASCPNTQLVYVGYSQGAQ   112
ApCut8          SCDYE--DILYYNAEGSDYCTA----VSEGDKNGIDQMTAYANKCPASKLVLTGYSQGAN   216
ApCut7          NCDYE--DILFYNPVESSFCAS----VEEGVANGIAQLSSYNARCPDTKLVISGYSQGSQ   116
ApCut4          GL-SQ--PILYPAGPAQN--------TSSGQEYLLHAISTGIQACPDQKYVLLGYSQGAS   65
AaCut2          GV-EY--DTIYPAGWSQI--------STQGTLDIVNKVQSTLRSDPDHCFVLEGYSQGAA   111
CS2Cle1         GT-IY--NTVYTADFSQN--------SAAGTADIIRRINSGLAANPNVCYILQGYSQGAA   123
ApCut1          GK-VY--NTVYAADYSQN--------SAAGTADIVNKITTTLRTSPNECFILEGYSQGAA   114
ApCut3          GT-VY--NTVYLADASQN--------SALGTQDIVNKVQSSLRTNPNMCFILQGYSQGAA   114
ApCut2          GT-EY--NTVYPASIDQN--------SAKGTADIINQITTGLKSNPNRCFILEGYSQGAA   116
FsCut1          GVWIQGVGGAYRATLGDNALP--RGTSSAAIREMLGLFQQANTKCPDATLIAGGYSQGAA   140
AoCutL          DVACQGVGPRYTADLPSNALP--EGTSQAAIAEAQGLFEQAVSKCPDTQIVAGGYSQGTA   130
ApCut6          -CSVQGVD--YPADAAGNANM-----GASGGPKMAALANQALKQCPDTKIILGGYSQGAM   147
ApCut5          NVATQGVT--YPADVAGAISGALNPGSAQGARTMASLTQQALSKCPDTKVILAGYSQGAE   145
BcCutA          SVTMNGVD--YPADVPGFLQG----GDPAGSQTMATMVTSTLSSCPDTKLVISGYSQGGQ   121
                          :                  .               *    :   *****
```

Supplementary Fig. S1 Alignment of amino acid sequences of known cutinases and ApCut1 to ApCut9. A red open box indicates a highly conserved GYSQG motif in the catalytic site. Pink-shaded residues show the position of a classical α/β-hydrolase catalytic triad Ser-His-Asp. Blue open boxes represent conserved cysteine residues that form disulfide bonds to maintain the conformation of the enzyme.

Supplementary Fig. S1 *(continued)*

```
ApCut9            IGDDALCGGGDPN-QG---ISSTAATISSSVGSKIKAVIFMGDPRQIPGASYSVGT-SK-   166
ApCut8            IAGDILGGADI------CAGLNPGLDPSTSPGNKIGAALIFGDNRHVADQSYNVLNGSS-   269
ApCut7            IVSDILGGGFGTFFQGCTTKPSPNLDVNSQVGKKVVAVTTFGNTRHTANQPYNQFSGSS-   175
ApCut4            LVLQASSR----------------L--GKKALEAIKAIILARNPYRIPGKSANVDSHGNT   107
AaCut2            ATVSALPK----------------L--TGDSFDAVKAVFLIGNPMHKSGLECNVDTLGGK   153
CS2Cle1           ATVVALQQ----------------LGTSGAAFNAVKGVFLIGNPDHKSGLTCNVDSNGGT   167
ApCut1            ATVNALPK----------------L--TGTANTAVKGVWLIGDPLHKAGLTCNIDSTGGT   156
ApCut3            ATVNAMPK----------------L--TGANMDAVKGVFLIGDPEHRSGLACNVDANGGT   156
ApCut2            ATVDAMSK----------------I--TGANFDAVKGVFLIGNPHHKSGLACNVDSNGGT   158
FsCut1            LAAASIED----------------L--DSAIRDKIAGTVLFGYTKNLQNR----------   172
AoCutL            VMNGAIKR----------------L--SADVQDKIKGVVLFGYTRNAQER----------   162
ApCut6            VVHNALNS----------------V--D---GSKIAAVTAFGDPMNGQ------------   174
ApCut5            QVHGALQN----------------L--Q---NGQVAVALTFGDPLQRM------------   172
BcCutA            LVHNAAKL----------------L--PAETTAKISSAVIFGDPDNGD------------   151
                                                    :          .

ApCut9            ----NPGFDPR-------PSGF--TCSAYASRIQAYCDAADPYCSNG-----NNAATHQG   208
ApCut8            ----VSSNDPR-------SPDSLARMNKFAGVLRSYCDQADPVCAAAG-PGPFTVDNHLN   317
ApCut7            ----GNGIFPR-------PAYQLANLATWTAKYHDYCVAEDPICAGG-----DNVEDHLN   219
ApCut4            DARGNIGMFVTQAITSNTPIPQFPESLGKSGKVLDYCLENDIVCASDPACDCQIAADHLS   167
AaCut2            STAYANGLEAYLG-----GI-----PDEWVSKTMDVCNFGDGVCDT--LTGIGITAQHLD   201
CS2Cle1           TTRNVNGLSVAYQ----GSV-----PSGWVSKTLDVCAYGDGVCDT--AHGFGINAQHLS   216
ApCut1            TTLNVNGISQYPGS---NSI-----PSGWISRTQDVCKYGDGVCDT--THGQGINAQHLS   206
ApCut3            TTKNVNGLSAVLG-----GI-----PAAWVPKTMDVCAYGDGVCDT--THGFGINAQHLS   204
ApCut2            TTKNVNGLTVLVG-----SI-----PANWVGKTLDVCAYGDGVCDT--AHGFGITAQHLS   206
FsCut1            -----------------GRIPN-----YPADRTKVFCNTGDLVCTG--S--LIVAAPHLA   206
AoCutL            -----------------GQIAN-----FPKDKVKVYCAVGDLVCLG--T--LIVAPPHFS   196
ApCut6            ------------------TFKG-----VDDSKVVRYCGSSDFVCDM--SGKTQGTGSHIS   209
ApCut5            ------------------PFRN-----IDSGRTKIYCNLGDGVCAG--A--FIISAAHLS   205
BcCutA            ------------------PVQG-----VSADRTDIICHAGDNICQG--G--SLILLAHLT   184
                                                      *   *  *             *

ApCut9            YGSEYGQAALKFVNSKLT------------------------------------------   226
ApCut8            YFDRYTDDAAGWVKYMLGY-----------------------------------------   336
ApCut7            YFDLYSEVAASWVKEQVKAADVVVSSTIVSPSSTFTSVATVPTDKAVPTTFSYSNNTATA   279
ApCut4            YGLVDSVQETAF-QHIVKVLA---------------------------------------   187
AaCut2            YPLDANVQKMGA-DFVVKALTS--------------------------------------   222
CS2Cle1           YPSDQGVQTMGY-KFAVNKLGGSA------------------------------------   239
ApCut1            YPNDATVQSMGA-KFVLGKLNA--------------------------------------   227
ApCut3            YPYSSTVQSMGT-KYMVAQLNGSS------------------------------------   227
ApCut2            YKSDANVQNQGI-KFVLGKLRGT-------------------------------------   228
FsCut1            YGPDARGPAPEFLIEKVRAVRGSA------------------------------------   230
AoCutL            YLSDT-GDASDFLLSQLG------------------------------------------   213
ApCut6            YGSNLAEAASRLAQ-IVGVKA---------------------------------------   229
ApCut5            YATQDATPAAQFAKGVIGNI----------------------------------------   225
BcCutA            YGMDTTAA-AAFVKKAAGL-----------------------------------------   202
                  *

ApCut9            ------------------------------------------------------------   226
ApCut8            ------------------------------------------------------------   336
ApCut7            PQVTNSAALSSIQSEVLPIPTADTTVTVTVDCASVDTSAIASWTSAFAATAVVSQSVAAS   339
ApCut4            ------------------------------------------------------------   187
AaCut2            ------------------------------------------------------------   222
CS2Cle1           ------------------------------------------------------------   239
ApCut1            ------------------------------------------------------------   227
ApCut3            ------------------------------------------------------------   227
ApCut2            ------------------------------------------------------------   228
FsCut1            ------------------------------------------------------------   230
AoCutL            ------------------------------------------------------------   213
ApCut6            ------------------------------------------------------------   229
ApCut5            ------------------------------------------------------------   225
BcCutA            ------------------------------------------------------------   202

ApCut9            ------------------------------------------------------- 226
ApCut8            ------------------------------------------------------- 336
ApCut7            ATGAAATTGSSGSKSSSAPNGTHTLMPYTGAASSMSGAVTGSLAIVLLGAFAMVL 394
ApCut4            ------------------------------------------------------- 187
AaCut2            ------------------------------------------------------- 222
CS2Cle1           ------------------------------------------------------- 239
ApCut1            ------------------------------------------------------- 227
ApCut3            ------------------------------------------------------- 227
ApCut2            ------------------------------------------------------- 228
FsCut1            ------------------------------------------------------- 230
AoCutL            ------------------------------------------------------- 213
ApCut6            ------------------------------------------------------- 229
ApCut5            ------------------------------------------------------- 225
BcCutA            ------------------------------------------------------- 202
```

Supplementary Fig. S2

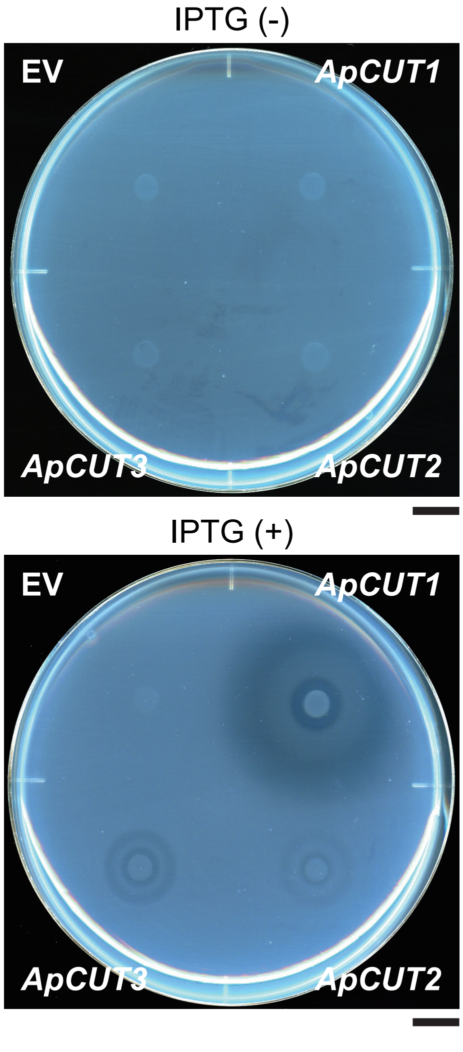

Supplementary Fig. S2 PCL-plate clearing assay of the extracts of ApCut1- to ApCut3-expressing *E. coli* cells. EV, empty vector. Bar, 1 cm.

Supplementary Fig. S3

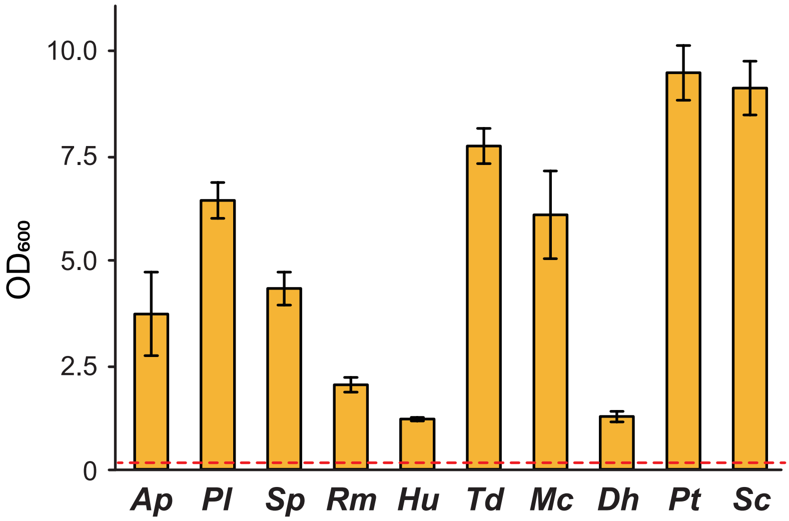

Supplementary Fig. S3 Growth of grape-skin residents and *S. cerevisiae* in YNB medium containing 10% (w/v) glucose as a sole carbon source. The graph indicates OD_{600} values after the 6-d fermentation test in YNB medium containing 10% (w/v) glucose. A red dashed line shows the initial OD_{600} value (OD_{600} = 0.1). Data represent mean values and standard deviations from three independent experiments. *Ap*, *A. pullulans*; *Pl*, *P. laurentii*; *Sp*, *S. pararoseus*; *Rm*, *R. mucilaginosa*; *Hu*, *H. uvarum*; *Td*, *T. delbrueckii*; *Mc*, *M. caribbica*; *Dh*, *D. hansenii*; *Pt*, *P. terricola*; *Sc*, *S. cerevisiae* X2180.

Supplementary Fig. S4

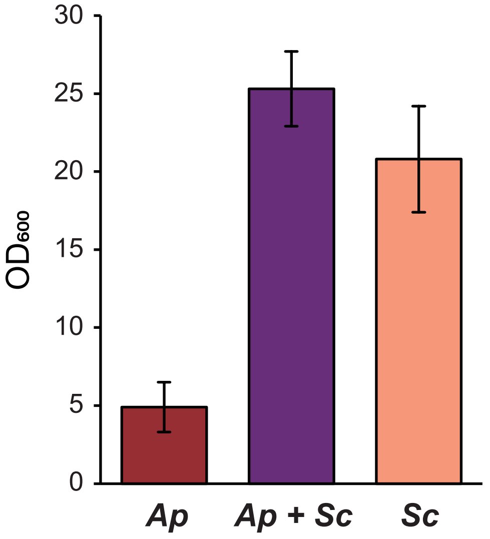

Supplementary Fig. S4 Growth in coculture of *A. pullulans* and *S. cerevisiae* in YNB medium containing 10% glucose as a sole carbon source. The graph indicates OD_{600} values after the 6-d fermentation test in YNB medium containing 10% (w/v) glucose. Data represent mean values and standard deviations from three independent experiments. *Ap*, inoculated with *A. pullulans* (red); *Ap* + *Sc*, coinoculated with *A. pullulans* and *S. cerevisiae* X2180 (violet); *Sc*, inoculated with *S. cerevisiae* X2180 (coral).

Supplementary Fig. S5

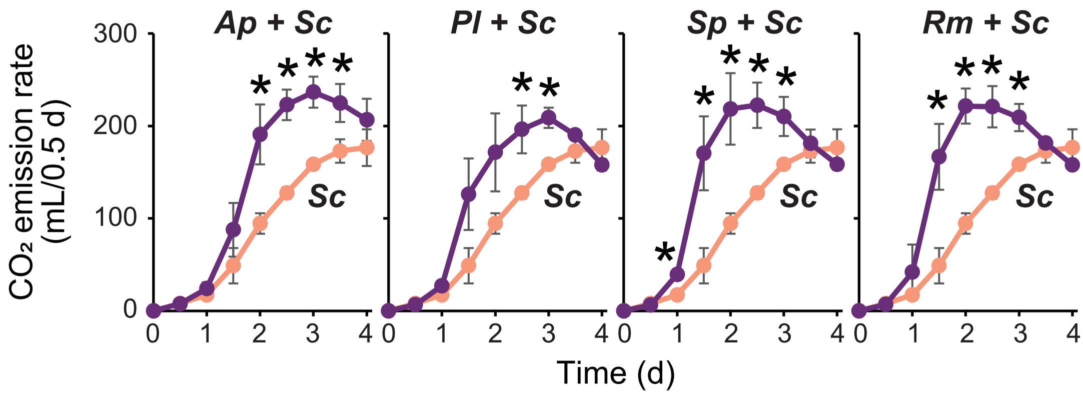

Supplementary Fig. S5 Alcoholic fermentation of intact grapes in coculture of grape-skin residents and *S. cerevisiae*. Carbon dioxide emission rates in a mixture of equal weight of YNB medium and intact grape berries are shown. Data represent mean values and standard deviations from two independent experiments. *Sc*, inoculated with *S. cerevisiae* X2180 (coral); *Ap* + *Sc*, coinoculated with *A. pullulans* and *S. cerevisiae* X2180, *Pl* + *Sc*, coinoculated with *P. laurentii* and *S. cerevisiae* X2180, *Sp* + *Sc*, coinoculated with *S. pararoseus* and *S. cerevisiae* X2180, *Rm* + *Sc*, coinoculated with *R. mucilaginosa* and *S. cerevisiae* X2180 (violet). Asterisks indicate statistically significant increases of carbon dioxide emission compared with *Sc* (t-test, $p < 0.05$).

Supplementary Fig. S6

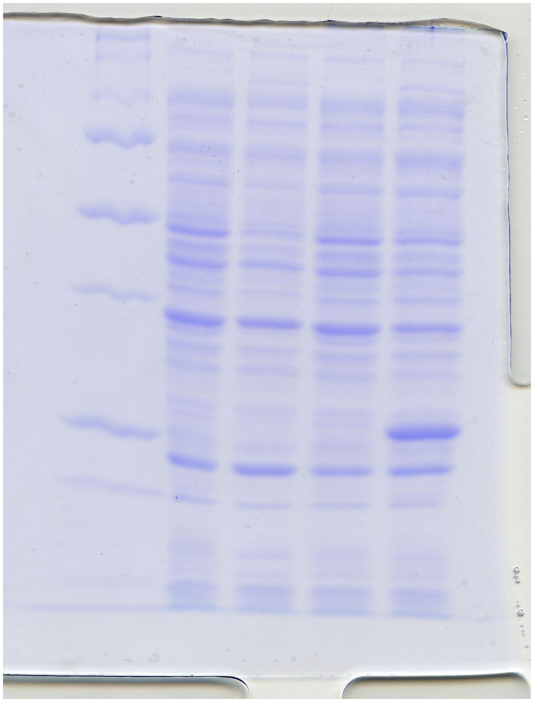

CBB staining

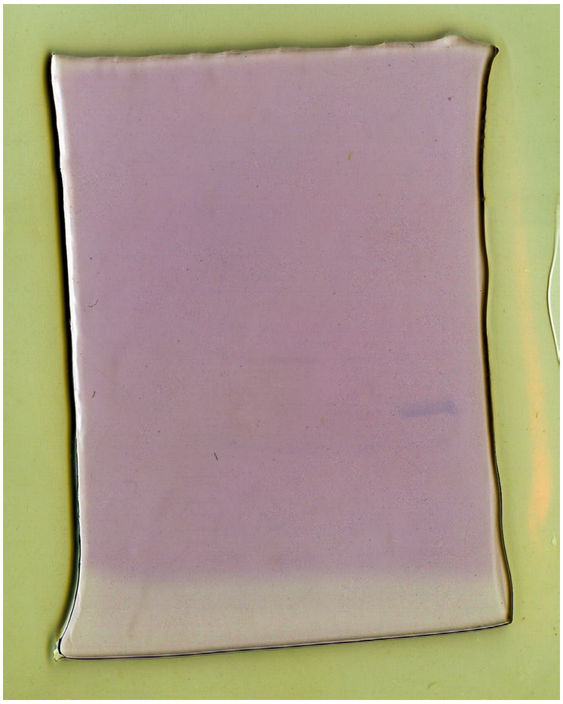

His-detect

Supplementary Fig. S6 Full-length gel images of Fig. 8b.

Supplementary Table S1 Yeasts or yeast-like microorganisms isolated from grapes in this study*.

No.	Grape variety	Grape species	Isolated from	Yeast species	Family	Category
1	Delaware	*Vitis* hybrid	Enrichment culture in water	*Aureobasidium pullulans*	Dothioraceae	Yeast-like fungus
**2**	**Delaware**	***Vitis* hybrid**	**Enrichment culture in water**	***Aureobasidium pullulans***	**Dothioraceae**	**Yeast-like fungus**
3	Delaware	*Vitis* hybrid	Enrichment culture in 5% sucrose	*Aureobasidium pullulans*	Dothioraceae	Yeast-like fungus
4	Delaware	*Vitis* hybrid	Enrichment culture in 5% sucrose	*Aureobasidium pullulans*	Dothioraceae	Yeast-like fungus
5	Delaware	*Vitis* hybrid	Enrichment culture in 5% sucrose	*Aureobasidium pullulans*	Dothioraceae	Yeast-like fungus
6	Delaware	*Vitis* hybrid	Surface-washed suspensions	*Sporobolomyces* sp.	Sporidiobolaceae	Basidiomycetous yeast
7	Delaware	*Vitis* hybrid	Surface-washed suspensions	*Sporobolomyces* sp.	Sporidiobolaceae	Basidiomycetous yeast
8	Delaware	*Vitis* hybrid	Surface-washed suspensions	*Aureobasidium pullulans*	Dothioraceae	Yeast-like fungus
9	Delaware	*Vitis* hybrid	Surface-washed suspensions	*Sporobolomyces* sp.	Sporidiobolaceae	Basidiomycetous yeast
10	Delaware	*Vitis* hybrid	Surface-washed suspensions	*Tremella yokohamensis* (*Cryptococcus yokohamensis*)	Tremellaceae	Basidiomycetous yeast
11	Delaware	*Vitis* hybrid	Surface-washed suspensions	*Tremella yokohamensis* (*Cryptococcus yokohamensis*)	Tremellaceae	Basidiomycetous yeast
12	Kyoho	*Vitis* hybrid	Juice (stem)	*Moesziomyces aphidis*	Ustilaginaceae	Basidiomycetous yeast
13	Kyoho	*Vitis* hybrid	Juice (stem)	*Moesziomyces aphidis*	Ustilaginaceae	Basidiomycetous yeast
14	Kyoho	*Vitis* hybrid	Juice (stem)	*Moesziomyces aphidis*	Ustilaginaceae	Basidiomycetous yeast
15	Kyoho	*Vitis* hybrid	Juice (stem)	*Moesziomyces aphidis*	Ustilaginaceae	Basidiomycetous yeast
16	Kyoho	*Vitis* hybrid	Juice	*Moesziomyces aphidis*	Ustilaginaceae	Basidiomycetous yeast
17	Kyoho	*Vitis* hybrid	Juice	*Moesziomyces aphidis*	Ustilaginaceae	Basidiomycetous yeast
**18**	**Kyoho**	***Vitis* hybrid**	**Juice**	***Sporidiobolus pararoseus* (*Sporobolomyces shibatanus*)**	**Sporidiobolaceae**	**Basidiomycetous yeast**
19	Kyoho	*Vitis* hybrid	Juice	*Sporidiobolus pararoseus* (*Sporobolomyces shibatanus*)	Sporidiobolaceae	Basidiomycetous yeast
20	Kyoho	*Vitis* hybrid	Juice	*Sporidiobolus pararoseus* (*Sporobolomyces shibatanus*)	Sporidiobolaceae	Basidiomycetous yeast
21	Kyoho	*Vitis* hybrid	Juice	*Sporidiobolus pararoseus* (*Sporobolomyces shibatanus*)	Sporidiobolaceae	Basidiomycetous yeast
22	Kyoho	*Vitis* hybrid	Juice	*Sporobolomyces* sp.	Sporidiobolaceae	Basidiomycetous yeast
23	Kyoho	*Vitis* hybrid	Juice	*Aureobasidium pullulans*	Dothioraceae	Yeast-like fungus
24	Kyoho	*Vitis* hybrid	Juice	*Aureobasidium pullulans*	Dothioraceae	Yeast-like fungus
**25**	**Shine Muscat**	***Vitis* hybrid**	**Juice**	***Rhodotorula mucilaginosa***	**Sporidiobolaceae**	**Basidiomycetous yeast**
26	Shine Muscat	*Vitis* hybrid	Juice	*Sporobolomyces* sp.	Sporidiobolaceae	Basidiomycetous yeast
27	Shine Muscat	*Vitis* hybrid	Juice	*Sporobolomyces* sp.	Sporidiobolaceae	Basidiomycetous yeast
28	Shine Muscat	*Vitis* hybrid	Juice	*Sporobolomyces* sp.	Sporidiobolaceae	Basidiomycetous yeast
29	Shine Muscat	*Vitis* hybrid	Juice	*Debaryomyces hansenii* (*Candida famata*)	Saccharomycetaceae	Ascomycetous yeast
**30**	**Shine Muscat**	***Vitis* hybrid**	**Juice**	***Debaryomyces hansenii* (*Candida famata*)**	**Saccharomycetaceae**	**Ascomycetous yeast**
31	Shine Muscat	*Vitis* hybrid	Juice	*Debaryomyces hansenii* (*Candida famata*)	Saccharomycetaceae	Ascomycetous yeast
32	Shine Muscat	*Vitis* hybrid	Surface-washed suspensions	*Sporobolomyces* sp.	Sporidiobolaceae	Basidiomycetous yeast
33	Shine Muscat	*Vitis* hybrid	Surface-washed suspensions	*Sporobolomyces* sp.	Sporidiobolaceae	Basidiomycetous yeast
34	Shine Muscat	*Vitis* hybrid	Surface-washed suspensions	*Papiliotrema aurea* (*Cryptococcus aureus*)	Tremellaceae	Basidiomycetous yeast
35	Shine Muscat	*Vitis* hybrid	Surface-washed suspensions	*Papiliotrema aurea* (*Cryptococcus aureus*)	Tremellaceae	Basidiomycetous yeast
36	Kyoho	*Vitis* hybrid	Surface-washed suspensions	*Sporobolomyces sp.*	Sporidiobolaceae	Basidiomycetous yeast
37	Kyoho	*Vitis* hybrid	Surface-washed suspensions	*Sporobolomyces sp.*	Sporidiobolaceae	Basidiomycetous yeast
38	Kyoho	*Vitis* hybrid	Juice	*Sporidiobolus pararoseus* (*Sporobolomyces shibatanus*)	Sporidiobolaceae	Basidiomycetous yeast
39	Shine Muscat	*Vitis* hybrid	Fermented juice	*Meyerozyma caribbica* (*Candida fermentati*)	Saccharomycetaceae	Ascomycetous yeast
40	Shine Muscat	*Vitis* hybrid	Fermented juice	*Meyerozyma caribbica* (*Candida fermentati*)	Saccharomycetaceae	Ascomycetous yeast
**41**	**Pinot noir**	***Vitis vinifera***	**Juice**	***Meyerozyma caribbica* (*Candida fermentati*)**	**Saccharomycetaceae**	**Ascomycetous yeast**
42	Pinot noir	*Vitis vinifera*	Juice	*Meyerozyma caribbica* (*Candida fermentati*)	Saccharomycetaceae	Ascomycetous yeast
**43**	**Pinot noir**	***Vitis vinifera***	**Juice**	***Pichia terricola***	**Saccharomycetaceae**	**Ascomycetous yeast**
44	Pinot noir	*Vitis vinifera*	Juice	*Pichia terricola*	Saccharomycetaceae	Ascomycetous yeast
**45**	**Pinot noir**	***Vitis vinifera***	**Juice**	***Hanseniaspora uvarum***	**Saccharomycetaceae**	**Ascomycetous yeast**
46	Pinot noir	*Vitis vinifera*	Juice	*Hanseniaspora uvarum*	Saccharomycetaceae	Ascomycetous yeast
47	Pinot noir	*Vitis vinifera*	Juice	*Hanseniaspora uvarum*	Saccharomycetaceae	Ascomycetous yeast
48	Pinot noir	*Vitis vinifera*	Juice	*Zygoascus meyerae* (*Candida hellenica*)	Saccharomycetaceae	Ascomycetous yeast
49	Pinot noir	*Vitis vinifera*	Fermented juice	*Pichia manshurica*	Saccharomycetaceae	Ascomycetous yeast
50	Pinot noir	*Vitis vinifera*	Fermented juice	*Hanseniaspora uvarum*	Saccharomycetaceae	Ascomycetous yeast
51	Pinot noir	*Vitis vinifera*	Fermented juice	*Hanseniaspora uvarum*	Saccharomycetaceae	Ascomycetous yeast
52	Pinot noir	*Vitis vinifera*	Fermented juice	*Hanseniaspora uvarum*	Saccharomycetaceae	Ascomycetous yeast
53	Pinot noir	*Vitis vinifera*	Fermented juice	*Meyerozyma caribbica* (*Candida fermentati*)	Saccharomycetaceae	Ascomycetous yeast
**54**	**Pinot noir**	***Vitis vinifera***	**Fermented juice**	***Torulaspora delbrueckii* (*Candida colliculosa*)**	**Saccharomycetaceae**	**Ascomycetous yeast**
55	Pinot noir	*Vitis vinifera*	Surface-washed suspensions	*Meyerozyma caribbica* (*Candida fermentati*)	Saccharomycetaceae	Ascomycetous yeast
56	Pinot noir	*Vitis vinifera*	Surface-washed suspensions	*Meyerozyma guilliermondii* (*Candida guilliermondii*)	Saccharomycetaceae	Ascomycetous yeast
57	Pinot noir	*Vitis vinifera*	Surface-washed suspensions	*Hanseniaspora uvarum*	Saccharomycetaceae	Ascomycetous yeast
58	Pinot noir	*Vitis vinifera*	Surface-washed suspensions	*Hanseniaspora uvarum*	Saccharomycetaceae	Ascomycetous yeast
59	Pinot noir	*Vitis vinifera*	Surface-washed suspensions	*Hanseniaspora uvarum*	Saccharomycetaceae	Ascomycetous yeast
60	Seto Giants	*Vitis* hybrid	Surface-washed suspensions	*Sporidiobolus pararoseus* (*Sporobolomyces shibatanus*)	Sporidiobolaceae	Basidiomycetous yeast
61	Seto Giants	*Vitis* hybrid	Fermented juice	*Rhodotorula paludigena*	Sporidiobolaceae	Basidiomycetous yeast
62	Seto Giants	*Vitis* hybrid	Fermented juice	*Rhodotorula paludigena*	Sporidiobolaceae	Basidiomycetous yeast
63	Muscat of Alexandria	*Vitis vinifera*	Fermented juice	*Trichosporon asahii*	Trichosporonaceae	Basidiomycetous yeast
64	Muscat of Alexandria	*Vitis vinifera*	Fermented juice	*Trichosporon asahii*	Trichosporonaceae	Basidiomycetous yeast
65	Gorbi	*Vitis* hybrid	Fermented juice	*Meyerozyma guilliermondii* (*Candida guilliermondii*)	Saccharomycetaceae	Ascomycetous yeast
66	Gorbi	*Vitis* hybrid	Fermented juice	*Meyerozyma guilliermondii* (*Candida guilliermondii*)	Saccharomycetaceae	Ascomycetous yeast
67	Pinot noir	*Vitis vinifera*	Surface-washed suspensions	*Hanseniaspora uvarum*	Saccharomycetaceae	Ascomycetous yeast
68	Pinot noir	*Vitis vinifera*	Surface-washed suspensions	*Hanseniaspora uvarum*	Saccharomycetaceae	Ascomycetous yeast
69	Pinot noir	*Vitis vinifera*	Surface-washed suspensions	*Hanseniaspora uvarum*	Saccharomycetaceae	Ascomycetous yeast
**70**	**Pinot noir**	***Vitis vinifera***	**Surface-washed suspensions**	***Papiliotrema laurentii* (*Cryptococcus laurentii*)**	**Tremellaceae**	**Basidiomycetous yeast**
71	Pinot noir	*Vitis vinifera*	Surface-washed suspensions	*Papiliotrema laurentii* (*Cryptococcus laurentii*)	Tremellaceae	Basidiomycetous yeast
72	Pinot noir	*Vitis vinifera*	Surface-washed suspensions	*Papiliotrema flavescens* (*Cryptococcus flavescens*)	Tremellaceae	Basidiomycetous yeast
73	Pinot noir	*Vitis vinifera*	Enrichment culture in water	*Hanseniaspora uvarum*	Saccharomycetaceae	Ascomycetous yeast
74	Pinot noir	*Vitis vinifera*	Enrichment culture in water	*Hanseniaspora uvarum*	Saccharomycetaceae	Ascomycetous yeast
75	Pinot noir	*Vitis vinifera*	Enrichment culture in water	*Papiliotrema laurentii* (*Cryptococcus laurentii*)	Tremellaceae	Basidiomycetous yeast
76	Pinot noir	*Vitis vinifera*	Enrichment culture in water	*Papiliotrema laurentii* (*Cryptococcus laurentii*)	Tremellaceae	Basidiomycetous yeast

*All clones shared >99% nucleotide identity in the rRNA gene ITS region, based on NCBI BLAST searches.
